# When polyuria does not stop: a case report on an unusual complication of hantavirus infection

**DOI:** 10.1186/s12879-020-05429-1

**Published:** 2020-09-29

**Authors:** Sebastian Schwab, Simon Lissmann, Niklas Schäfer, Alexander Isaak, Dietrich Klingmüller, Ulrike Attenberger, Anna M. Eis-Hübinger, Jörg Hofmann, Christian P. Strassburg, Philipp Lutz

**Affiliations:** 1 Department of Internal Medicine, Faculty of Medicine, University Bonn, Bonn, Germany; 2 Institute of Experimental Immunology, Faculty of Medicine, University Bonn, Bonn, Germany; 3 Department of Neurology, Faculty of Medicine, University Bonn, Bonn, Germany; 4 Department of Radiology, Faculty of Medicine, University Bonn, University of Bonn, Bonn, Germany; 5 Institute of Virology, Faculty of Medicine, University Bonn, University of Bonn, Bonn, Germany; 6 Freie Universität Berlin, Humboldt-Universität zu Berlin, and Berlin Institute of Health, Institute of Virology, Charité – Universitätsmedizin Berlin, Berlin, Germany

**Keywords:** Central diabetes insipidus, Hantavirus infection, Pituitary hemorrhage, Case report

## Abstract

**Background:**

The clinical features, course and outcome of hantavirus infection is highly variable. Symptoms of the central nervous system may occur, but often present atypically and diagnostically challenging. Even though the incidence of hantavirus infection is increasing worldwide, this case is the first to describe diabetes insipidus centralis as a complication of hantavirus infection in the Western world.

**Case presentation:**

A 49-year old male presenting with severe headache, nausea and photophobia to our neurology department was diagnosed with acute haemorrhage in the pituitary gland by magnetic resonance imaging. In the following days, the patient developed severe oliguric acute kidney failure. Diagnostic workup revealed a hantavirus infection, so that the pituitary haemorrhage resulting in hypopituitarism was seen as a consequence of hantavirus-induced hypophysitis. Under hormone replacement and symptomatic therapy, the patient’s condition and kidney function improved considerably, but significant polyuria persisted, which was initially attributed to recovery from kidney injury. However, water deprivation test revealed central diabetes insipidus, indicating involvement of the posterior pituitary gland. The amount of urine production normalized with desmopressin substitution.

**Conclusion:**

Our case report highlights that neurological complications of hantavirus infection should be considered in patients with atypical clinical presentation.

## Background

Hantavirus is a zoonotic viral infection, which is transmitted via aerosols of rodents, shrews and bats excrements to humans. Of more than fourty known species of hantavirus, twenty two are considered pathogenic by causing different syndromes such as hemorrhagic fever with kidney syndrome (HFRS), which is mainly encountered in Eurasia, or hantavirus cardiopulmonary syndrome (HCPS) that predominantly occurs in South and North America [1–5].

The clinical features, course and outcome of hantavirus infection is highly variable, depending on the virus strain. Symptomatic hantavirus infection with the Puumala strain typically manifests with an abrupt onset of fever, headache, body aches, thrombopenia, renal impairment, and in a minor proportion with neurological symptoms [6].

Over the last years, hantavirus infections are on the rise and affect approximately 200,000 humans annually worldwide [1, 5].

Impairment of the posterior pituitary clinically presents as diabetes insipidus, which is characterized by hypotonic polyuria due to attenuated arginine vasopressin- (AVP) secretion. The condition can be inherited or acquired by trauma, infection or autoimmune disorders [7]. Besides the well appreciated affection of the kidney, symptoms of the central nervous system (CNS) may occur, but may present atypically and complex [1, 4]. Few case reports and case series about hypopituitarism in hantavirus infection have been published over the past decades [8–11]. However, we believe that pituitary malfunction accompanying hantavirus infection is not well known in everyday clinical practice. In contrast to anterior pituitary involvement, there is only one report from a Korean patient describing dysfunction of the posterior pituitary gland [12].

Here we report a case of a hantavirus associated pituitary bleeding following an infection by Puumala virus, the etiological agent of the most common hantavirus infection in Europe. Pituitary haemorrhage led to a global dysfunction of both anterior and posterior pituitary gland resulting in insufficiency of the corticotropic, thyreotropic and gonadotrophic axis as well as in central diabetes insipidus.

## Case presentation

A 49-year old male patient self-reported to the hospital with strong piercing headaches, which were initially located at the front, but spread occipitally later, and were accompanied by photophobia. For the three previous days, the patient had suffered from severe nausea, vomiting, diarrhea and general sickness. The patient presented with normothermia, normal vital signs, no altered level of consciousness and a Glasgow Coma Scale of 15. Trauma or drug intoxication could be excluded by the history of the patient.

**Fig. 1:**
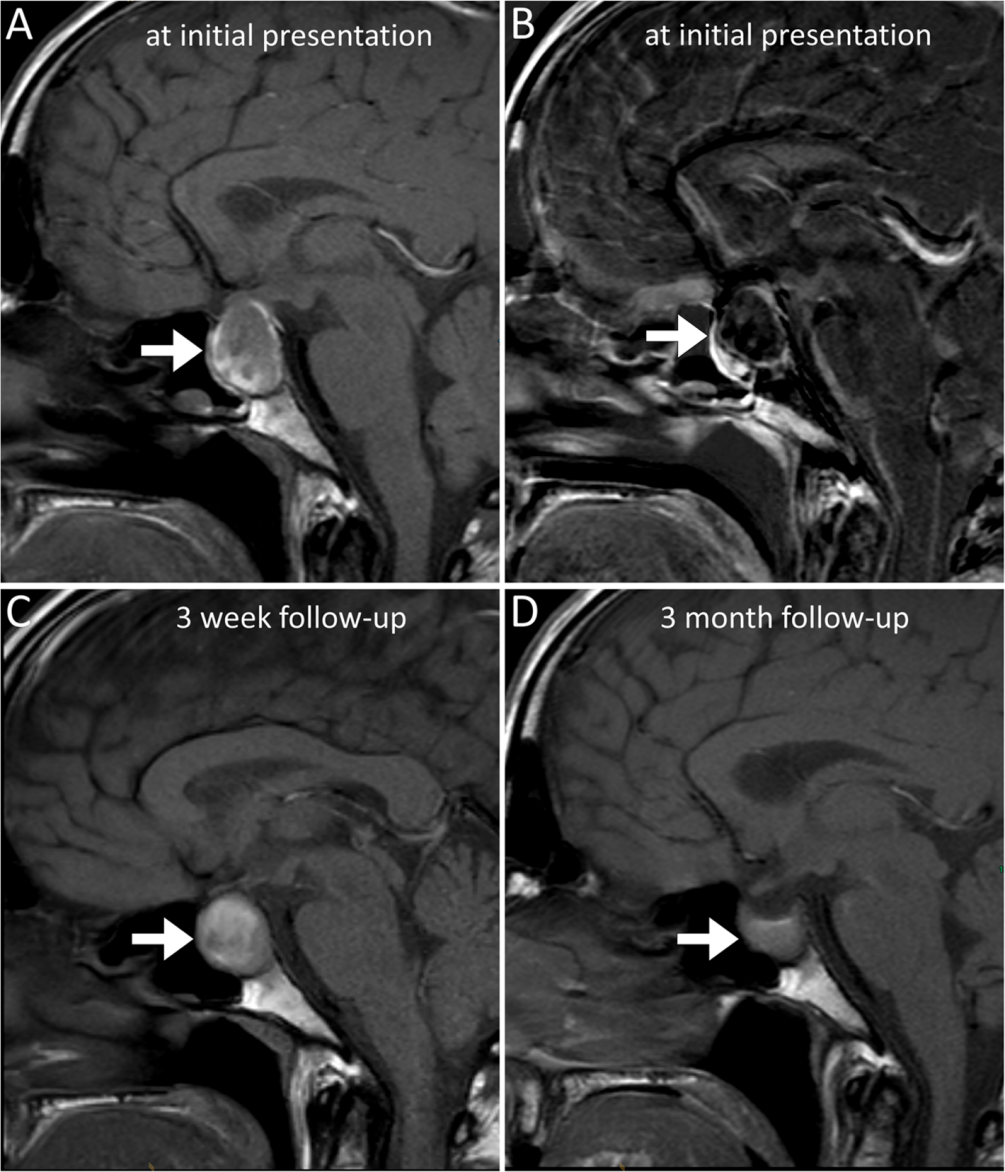
Magnetic resonance imaging of the sellar region. Magnetic resonance imaging (MRI) of the head disclosed a hemorrhagic intrasellar mass (arrow) with compression of the optic chiasm on initial presentation (**a**, native T1-weighted sequence in sagittal orientation shows a hyperintense signal in the periphery of the mass due to blood. **b**, subtraction of native and contrast-enhanced T1-weighted sequences shows only minimal peripheral and central enhancement). Follow-up MRI shows a shrinking and organized sellar hematoma after 3 weeks (**c**, native T1-weighted sequence) and 3 months, respectively (**d**, native T1-weighted sequence)

After clinical and laboratory examination a computed tomography scan was initiated and showed a mass in the sellar region. Subsequently, magnetic resonance imaging (MRI) was performed and revealed a haemorrhagic sellar mass, thus, the diagnosis of subacute pituitary apoplexy was made (Fig. 1).

Laboratory workup on day five after admission showed that pituitary apoplexy was associated with insufficiency of the corticotropic, thyrotropic and gonadotrophic axis. Although a shift of the optic chiasm could be shown by the MRI scan, the ophthalmological examination showed only minor non-specific defects in the visual field but neither papilledema nor orthoptic abnormalities.

**Fig. 2:**
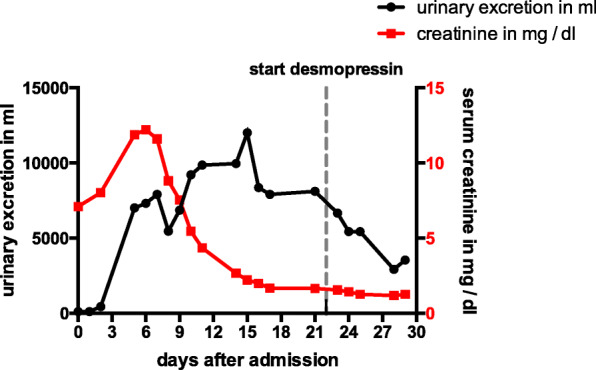
Kidney function and urinary excretion over time. Level of serum-creatine (per mg / dl) as surrogate parameter for kidney function as well as urinary excretion (per ml). On day 22 after admission, desmopressin treatment was initiated and urinary excretion decreased

Based on the radiological findings, a pituitary apoplexy caused by haemorrhage of a hypophyseal macroadenoma was suspected. Surgical intervention was planned, but postponed when laboratory follow-up revealed severe acute kidney injury with a creatinine of 11.6 mg / dl and an estimated glomerular filtration rate (eGFR) < 20 ml/min on day 5 after admission (Fig. 2).

The combination of kidney failure, thrombocytopenia and neurological symptoms prompted us to consider hantavirus-infection. In line with this possibility, the patient, who worked as a driving instructor, reported on digging out a bamboo hedge prior to hospitalization.

**Fig. 3:**
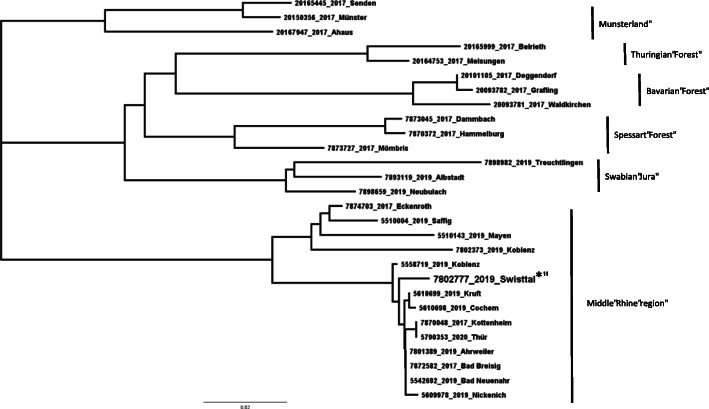
Phylogenetic analysis of patient‘s puumalavirus. Maximum likelihood (ML) phylogenetic tree of partial L-Segments of orthohantaviruses based on 347 nt alignment calculated using the GTR + G model of nucleotide substitution. The tree was calculated with PhyML3.0 [13] using the best-fitting model according to Smart Model Selection as implemented on the website, and 1000 bootstrap (BS) replicates. The patient’s puumala sequence is labeled with *. For reason of clarity and comprehensibility only few strains of all known outbreak regions in Germany were depicted. The scale bar represents the percentage of substitutions per site

Further testing on day three after admission revealed hantavirus infection by detection of IgM and IgG antibodies against Puumala hantavirus using immunofluorescence assays and line immunoassays. Polymerase chain reaction for hantavirus was also positive. Specific amplification and subsequent sequencing of the partial L-Segment revealed an infection with a puumalavirus that circulates in the Middle Rhine region in Germany (Fig. 3). The detection of IgM and viral RNA strongly suggested acute hantavirus infection. Therefore, we did not assess changes of antibody titers over time. In addition, infection by hepatitis B virus, hepatitis C virus and HIV was ruled out by serological testing. Because testing for hantavirus yielded positive results, we did not test for leptospirosis, which is quite rare in Germany in the absence of occupational risk factors.

In the meantime, the patient had been transferred to the nephrology department. He became oliguric. Since he complained of increasing nausea in association to rising blood urea levels, we considered haemodialysis, but kidney function started to recover in time to avoid renal replacement therapy. After symptomatic treatment and hormonal replacement, urinary excretion increased on day 3 and creatinine levels decreased from day 6 onwards (Fig. 2). No specific antiviral treatment was applied.

Although the patient’s condition improved largely, diuretic medication was withheld from day 5 and a negative fluid balance with continuous weight loss was achieved every day, there was no decline in urine excretion (urinary volume 6.25 ml/kg/h) (Fig. 2).

Initially, this was attributed to the polyuric phase of recovery from acute kidney injury. Since the plasma hyperosmolality associated with urine hypoosmolality persisted, central diabetes insipidus was suspected and a water deprivation test was carried out. Based on copeptin levels during thirsting (copeptin t0–4.3 pmol/l; copeptin t7–4.2 pmol/l) and response to desmopressin, we diagnosed central diabetes insipidus (Table 1). The polyuria of up to 12 L / d was normalized during the following days by application of desmopressin.

**Table 1:** Water deprivation test

	Urine		Plasma			Vital signs	
Hour	Volume [ml/h]	Osmolality [mosmol/kg]	Osmolality [mosmol/kg]	Sodium [mmol/l]	Copeptin [pmol/l]	Bodyweight [kg]	Blood Pressure [mmHg]
**0**	430	164	299	144	4.3	91.2	140/80
**1**	300	157	298	144		n/a	n/a
**2**	350	161	299	144		90.1	130/80
**3**	320	197	300	147		89.7	n/a
**4**	300	208	304	148		89.4	150/90
**5**	250	223	304	147		89.6	130/70
**6**	250	231	305	148		89.3	140/80
**7**	300	238	308	148	4.2	88.9	130/75
**Administration of 4** μg **desmopressin (Minirin®)**							
**8**		387					
**9**		301					

Low Copeptin levels and a > 50% increase of urine osmolality after administration of desmopressin confirmed the diagnosis of a complete diabetes insipidus centralis

The patient was discharged with substitution of L-thyroxine 100 μg per day, hydrocortisone (15–10-0 mg), desmopressin 10 μg bid and testosterone depot 250 mg.

Follow-up MRI after 3 weeks showed that the hemorrhage was consolidating with a regressive shift of the chiasma opticum (Fig. 1).

When seen as outpatient in our endocrinological department at 6 – month follow-up, the patient reported to be in good general condition. Laboratory parameters were unremarkable except for a reduced serum level of LH (0.4 U/l; normal range: 1.7–8.6). Given the partial recovery of pituitary function at the 6-month follow-up, dosage of desmopressin and of hydrocortisone were reduced. Thyroid and testosterone replacement was continued unchanged.

## Discussion and conclusion

Here, we present a case of global pituitary insufficiency and central diabetes insipidus due to hantavirus infection. Despite pituitary bleeding and renal failure, our patient did not meet the classical criteria of haemorrhagic fever with renal syndrome (HFRS), because he did neither present with fever nor with haemorrhagic skin lesions [14]. Most likely, pituitary bleeding affected the pituitary stalk rather than the posterior pituitary directly. Another possibility would be that posterior pituitary dysfunction resulted from replacement of the injured stalk by fibrous connective tissue due to the severe anterior pituitary damage, as previously reported [15]. However, the significant improvement during follow-up renders this scenario very unlikely.

Cases of pituitary haemorrhage and hypopituitarism associated with hantavirus infection have been reported previously [6, 9, 16, 17]. Direct viral infection of the pituitary gland has been proven histologically in one of these cases [8]. One study aiming to determine the prevalence of hypopituitarism among hantaviral HFRS survivors found that 11 of the 60 patients (18%) showed deranged hormonal levels [18]. Another, older study of autopsies in patients after lethal HFRS revealed that 72% of the patients had anterior pituitary necrosis [19]. A more recent study reported two patients (3.4%) with bleeding in the pituitary gland out of a cohort of 58 patients with HFRS due to infection with Puumala virus. Interestingly, the follow-up investigation of these two patients did not reveal any late-onset pituitary insufficiency [20].

Whereas affection of the anterior pituitary by hantavirus is not uncommon, there is only one report about hantavirus associated affection of posterior pituitary function from a Korean patient with HFRS, resulting in panhypopituitarism and central diabetes insipidus [12]. Sellar MRI in this patient, however, was normal, indicating that affection of the posterior pituitary gland may be a direct effect of hantavirus infection.

Hantavirus infections clinically present very similar to leptospirosis, another emerging bacterial zoonosis [4, 21]. Leptospirosis infections are more common worldwide than Hantavirus infections with high incidence in tropical and subtropical areas [21]. Serology and PCR, are required to distinguish between these two entities, but are often unavailable, hampering identification of the correct diagnosis.

In addition, had this case happened in a developing country and the patient had gone to a healthcare facility without CT / MRI scanner, the treating doctors would have been compelled to rely only upon clinical signs and thus might have missed the diagnosis of pituitary hemorrhage. This is an example of the importance for a wider accessibility of brain imaging facilities to vulnerable populations [22].

In the case of our patient, hantavirus infection most likely lead to a haemorrhagic pituitary apoplexy due to hantavirus-induced hypophysitis. Whether affection of the posterior pituitary gland was related to the infection itself or was secondary to the bleeding cannot be distinguished. Possibly, a preexisting, so far asymptomatic adenoma may have favored the hemorrhage in the context of the hypophysitis and affected hormonal function negatively once the intrasellar pressure increased due to the bleeding.

Commonly, one of the characteristics of hantavirus infection is thrombocytopenia [23] and a deficit in functional platelets, which favours intracranial haemorrhage in more severe cases. An increase in capillary permeability is common, which is attributed to viral infection of endothelial cells impairing both the endothelial barrier and the cell function. The interaction of platelets with the infected endothelium may also be important [24], but the limited availability of animal models hampers further in-depth investigation.

Importantly, our case report points out that cerebral symptoms along with acute kidney injury should raise the suspicion of hantavirus infection, especially in months when hantavirus infections usually occur.

The extent to which anterior pituitary insufficiency after hantavirus-associated pituitary bleeding recedes is not known due to the rarity of this complication, but the published cases indicate that a full recovery may occur [20]. The prognosis of malfunction of the posterior pituitary after hantavirus infection is unknown and should be studied in the future. However, the so far at least partial functional recovery of the anterior and posterior pituitary gland in our patient indicates that not only prognosis of hantavirus-induced hypopituitarism, but also of diabetes insipidus may be favorable.

Taken together, our report underscores that clinicians should be aware of hypopituitarism as potential complication of hantavirus infection and should assess for hormonal deficiency in case of clinical suspicion, as hormone replacement might be life-saving. We report diabetes insipidus centralis as an important complication, which is diagnostic challenging, because a polyuric phase is common in hantavirus-induced acute kidney failure.

## Data Availability

The datasets used and/or analysed during the current study are available from the corresponding author on reasonable request.

## Abbreviations

AVP: Arginine vasopressin
CNS: Central nervous system
IgM: Immunglobulin M
IgG: IMMUNGLOBULIN G
LH: *Luteinizing hormone*
MRI: Magnetic resonance imaging
HFRS: Haemorrhagic fever with renal syndrome
HCPS: Hantavirus cardiopulmonary syndrome
